# Mitigating alkaline instability induced by tyrosine–tyrosine repulsion in an FcγRIIIa-binding protein through phenylalanine substitution

**DOI:** 10.1093/jb/mvag023

**Published:** 2026-03-13

**Authors:** Rio Okuda, Yuki Tokunaga, Satoru Nagatoishi, Ryo Matsunaga, Yosuke Terao, Teruhiko Ide, Kouhei Tsumoto

**Affiliations:** Department of Bioengineering, School of Engineering, The University of Tokyo, 7-3-1 Hongo, Bunkyo-ku, Tokyo 113-8656, Japan; Department of Bioengineering, School of Engineering, The University of Tokyo, 7-3-1 Hongo, Bunkyo-ku, Tokyo 113-8656, Japan; Department of Bioengineering, School of Engineering, The University of Tokyo, 7-3-1 Hongo, Bunkyo-ku, Tokyo 113-8656, Japan; Medical Device Development and Regulation Research Center, School of Engineering, The University of Tokyo, 7-3-1 Hongo, Bunkyo-ku, Tokyo 113-8656, Japan; Department of Bioengineering, School of Engineering, The University of Tokyo, 7-3-1 Hongo, Bunkyo-ku, Tokyo 113-8656, Japan; Life Science Research Laboratory, Tosoh Corporation, 2743-1 Hayakawa, Ayase, Kanagawa 252-1123, Japan; Life Science Research Laboratory, Tosoh Corporation, 2743-1 Hayakawa, Ayase, Kanagawa 252-1123, Japan; Department of Bioengineering, School of Engineering, The University of Tokyo, 7-3-1 Hongo, Bunkyo-ku, Tokyo 113-8656, Japan; Medical Device Development and Regulation Research Center, School of Engineering, The University of Tokyo, 7-3-1 Hongo, Bunkyo-ku, Tokyo 113-8656, Japan; The Institute of Medical Science, The University of Tokyo, 4-6-1 Shirokanedai, Minato-ku, Tokyo 108-8639, Japan

**Keywords:** alkaline stability, aromatic substitution, clean-in-place resistance, Fc gamma receptor IIIa, protein engineering, tyrosine deprotonation, Y59F mutation

## Abstract

Proteins are essential components in biotechnological and biopharmaceutical applications; however, their structural instability under alkaline conditions presents significant limitations. High-pH environments, such as chromatographic clean-in-place (CIP) protocols, frequently cause protein degradation and loss of biological activity. Current strategies for engineering alkali-stable proteins include rational design approaches targeting deamidation-susceptible residues, surface charge optimization and enzyme extraction from alkaliphilic organisms. However, the fundamental principles governing alkaline stability remain poorly understood. In this study, we investigated alkaline stability mechanisms in Fc gamma receptor IIIa, a critical immune effector protein with applications in antibody purification and glycoform analysis. Systematic mutagenesis identified a tyrosine-to-phenylalanine substitution at position 59 that significantly enhanced protein stability during alkaline CIP exposure while retaining substantial IgG binding activity. Structural and biophysical characterizations revealed that this substitution prevents the deprotonation of tyrosine that occurs at alkaline pH, thereby mitigating destabilizing electrostatic repulsion within the protein structure. Our findings support a model in which targeted aromatic substitution enhances alkaline stability without severely compromising protein function and provide mechanistic insight into the contribution of buried tyrosine ionization to alkaline instability in FcγRIIIa.

## Graphical Abstract

**Graphical Abstract:**
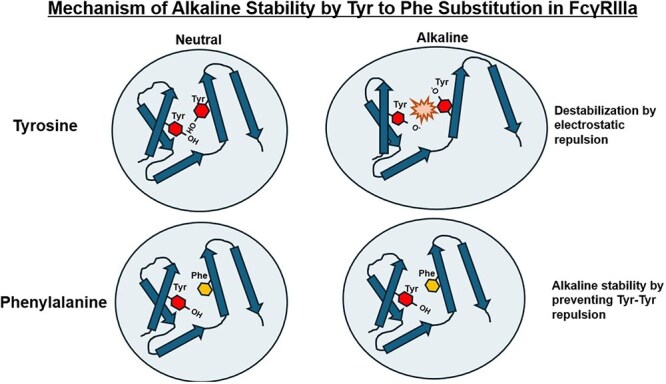

Proteins are essential biomolecules for various industrial applications, including biopharmaceuticals, enzyme-based catalysis and biosensors *(**1**,*  *2**)*. Many of these applications require proteins to maintain their functionality and structural stability under harsh conditions, including alkaline conditions, such as those encountered in detergent formulations, industrial bioprocessing and therapeutic applications *(**3**,*  *4**)*. However, most proteins are inherently unstable at high pH values, leading to structural unfolding and loss of functional activity. This instability limits their industrial utility, highlighting the need to develop proteins that can withstand alkaline conditions while retaining their structural integrity and functional activity *(**5**–**7**)*.

Previous studies have demonstrated several successful strategies for the development of alkali-stable proteins. One approach involves identification and modification of deamidation-prone residues, particularly asparagine and glutamine, via site-directed mutagenesis. This strategy was successfully applied to protein A, where a G29A mutation in the B domain prevents deamidation of a susceptible Glu–Gly sequence *(**8**)*. Another rational design strategy focuses on modifying surface-exposed charged residues to minimize electrostatic repulsion under alkaline conditions. For instance, Shirai *et al.* reported that substituting negatively charged residues (aspartic acid and glutamic acid) with arginine, together with neutral hydrophilic residues, significantly improves the alkaline stability of M-protease *(**9**)*. Collectively, these rational design studies provide valuable mechanistic insights into how specific amino acid substitutions enhance protein stability under high pH conditions.

Another strategy involves sourcing proteins from alkaliphilic organisms that have evolved naturally to maintain stability and function under high pH conditions *(**10**)*. Laccase has attracted considerable attention as a biocatalyst with potential applications in diverse industries. However, the stability and catalytic activity of conventional laccases are highly sensitive to pH variations, limiting their industrial utility. To address this limitation, researchers have investigated key mutations in laccases produced by the alkaliphile *Bacillus subtilis*. Comparative analyses revealed that the substitutions V110E and S427Q are critical mutations that are essential for sustaining catalytic activity under alkaline conditions *(**11**)*.

Despite these advances, relatively few proteins have been systematically engineered for alkaline resistance using rational design. A deeper understanding of the molecular basis of stability under alkaline conditions is vital for establishing broadly applicable engineering principles.

This study investigated the molecular basis of alkaline stability involving Fc gamma receptor IIIa (FcγRIIIa). FcγRIIIa is a key immune receptor that mediates antibody-dependent cellular cytotoxicity and is instrumental in the efficacy of therapeutic antibodies *(**12**–**14**)*. Recently, a protein ligand-based chromatography method has been developed utilizing immobilized engineered FcγRIIIa to separate IgGs according to their glycosylation profiles *(**15**,*  *16**)*.

During the development of this chromatography system, targeted mutagenesis was performed on FcγRIIIa to enhance its stability under alkaline conditions. The substitution of tyrosine at position 59 with phenylalanine (Y59F) significantly improved stability in high-pH environments, enabling the receptor to withstand clean-in-place (CIP) alkaline washes while retaining its IgG-binding activity. Among the tyrosines in FcγRIIIa, Y59 and Y72 form the only buried, closely positioned aromatic pair, creating a distinctive aromatic cluster within the hydrophobic core. We propose that alkaline sensitivity originates when these buried tyrosines deprotonate at high pH, generating phenolate species that introduce unfavourable electrostatics interactions and destabilize the core. Because of their spatial proximity, Y59 and Y72 are the only residues capable of producing such pH-dependent repulsion upon simultaneous deprotonation. Initially, both tyrosine residues were considered candidates for modification. However, Y72 plays a crucial role in stabilizing the overall structure of FcR (unpublished data), whereas Y59 is structurally resistant to substitution. Therefore, this study focuses on Y59.

Direct experimental evidence for functional relevant tyrosine deprotonation in proteins remains limited. For example, previous study done by Ishikita and Saito used structure-based pKa analyses to show that deprotonation of a buried tyrosine can induce local hydrogen-bond rearrangements and side-chain displacement *(**17**)*. By characterizing Y59 and the Y59F variant in FcγRIIIa, we aim to clarify whether buried-tyrosine ionization influences local conformation and Fc binding and whether phenylalanine substitution can preserve stability and function at elevated pH.

## Results

### Comparative binding analysis of FcγRIIIa mutants by enzyme-linked immunosorbent assay after alkaline treatment

To evaluate the effect of alkaline stress on binding activity, an enzyme-linked immunosorbent assay (ELISA) was performed to compare the binding of parent-type FcγRIIIa (PT) and Y59F mutant to trastuzumab before and after alkaline treatment. Alkaline-treated samples were prepared by incubating 4 μM protein in 50 mM NaOH at 30°C for 90 min, whereas untreated controls were prepared under identical conditions using buffer without NaOH. Following treatment, all samples were diluted 4-fold in buffer (50 mM sodium acetate, pH 5.5) to a final concentration of 1 μM. Subsequently, ELISA was performed to assess the ability of both untreated and alkaline-treated samples to bind immobilized trastuzumab.

**Fig. 1:**
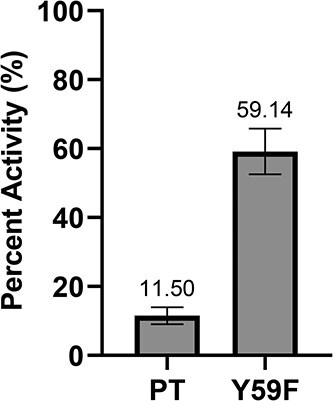
ELISA comparing binding activity with or without NaOH treatment.

The untreated Y59F samples exhibited lower absorbance, indicating a slight loss in binding (Supplementary Table S1). The binding activity was quantified as residual activity, defined as the ratio of the absorbance of the alkaline-treated sample to that of the untreated control. The PT retained only 11.50 ± 2.48% of its binding activity following alkaline treatment, whereas the Y59F mutant retained 59.14 ± 6.62% (Fig. 1, Supplementary Table S1). These results confirmed that the Y59F substitution is a contributing factor in maintaining activity under alkaline conditions, thereby preserving its functional binding capacity.

### Surface plasmon resonance analysis of binding kinetics before and after alkaline treatment

**Fig. 2:**
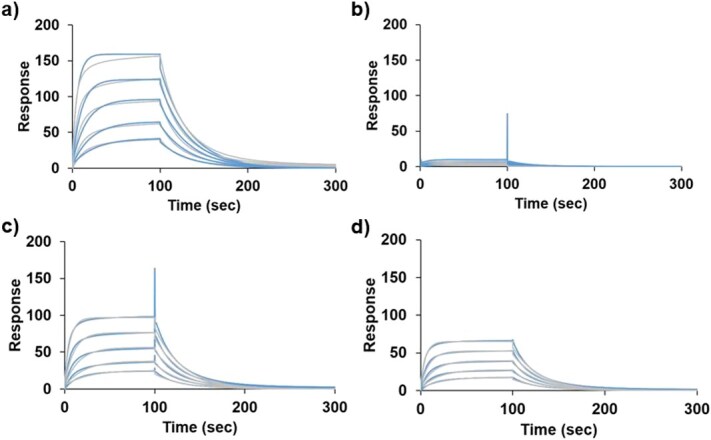
SPR comparing binding activity before and after alkaline treatment: (A) PT non-treated, (B) PT alkaline treated, (C) Y59F non-treated and (D) Y59F alkaline treated. Grey lines represent the raw sensorgram, and the blue lines represent the fitting curve generated by the Biacore analysis software.

To characterize the kinetic basis for the ELISA results, we measured real-time binding of PT and Y59F to trastuzumab by surface plasmon resonance (SPR). Biotinylated FcγRIIIa was captured on the streptavidin sensor chip and trastuzumab was injected as the analyte. Sensorgram for untreated PT and Y59F showed similar kinetics, with fitted *K*_D_ values of 90.1 ± 1.1 and 100.2 ± 0.7 nM, respectively, and comparable *k*_on_ and *k*_off_ (Supplementary Table S2). However, the *R*_max_ of Y59F was lower, suggesting that slight loss in binding was observed. After alkaline treatment, Y59F exhibited a similar *K*_D_ for the binding fraction that remained active, but it showed a reduced maximum response, indicating that a portion of the immobilized ligand no longer binds to trastuzumab. Alkaline-treated PT showed unreliable binding response (Fig. 2A–D). Together with the ELISA data, these results indicate that the Y59F retains a larger fraction of functional binder after alkaline stress and is therefore consistent with enhanced functional resistance to alkaline stress.

### Exploring the potential contribution of aggregation, hydrolysis and chemical modifications to protein degradation under alkaline conditions

Under alkaline conditions, proteins are prone to degradation and exhibit increased rates of aggregation and hydrolysis *(**18**–**20**)*. Furthermore, various chemical modifications such as deamidation, β-elimination and backbone cleavage are viable options *(**21**,*  *22**)*. For tyrosine, we specifically assessed whether phenolic side chains could be converted to quinone or catechol derivatives *(**23**)*. To investigate whether FcγRIIIa undergoes chemical modification, aggregation or hydrolysis under alkaline conditions, we performed dynamic light scattering (DLS), analytical size-exclusion chromatography (SEC), size exclusion chromatography–multi-angle light scattering (SEC-MALS), UV spectroscopy, SDS-PAGE with Coomassie Brilliant Blue (CBB) staining and western blotting.

DLS (UNcle) of FcγRIIIa at pH 5.5 and 12.5 showed lower amplitudes and slightly broader mass/intensity peaks at pH 12.5, with minor >100 nm peaks in PT (pH 5.5) and Y59F (pH 12.5). SEC/SEC-MALS yielded a single peak at both pH values, and SDS-PAGE/western blot showed single bands of similar intensity, indicating no extensive hydrolytic degradation and only limited soluble aggregation/heterogeneity (Supplementary Fig. S1A–L).

Soluble concentrations of PT and Y59F (1.0 mg/ml) were unchanged after incubation at pH 5.5 or 12.5 for up to 90 min, indicating that Y59F’s improved alkaline stability is not due to reduced precipitation (Supplementary Table S3). UV spectroscopy showed no tyrosine modification: for PT and Y59F at both pH values, the 280-nm absorbance was unchanged and no new peaks appeared above 300 nm, indicating no detectable quinone or catechol formation (Supplementary Fig. S1M and N).

### Circular dichroism comparison of PT and Y59F FcγRIIIa

To further characterize the structural effects of alkaline treatment, Y59F mutants were incubated in 50 mM NaOH at 30°C for 90 min, and circular dichroism (CD) spectroscopy was performed to evaluate changes in secondary structure. The obtained spectra were compared with those of untreated controls.

**Fig. 3:**
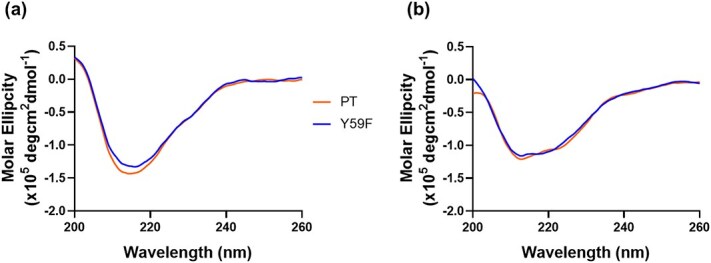
CD measurement comparison between PT and Y59F at (A) pH 5.5 (left) and (B) pH 12.5 (right).

At pH 5.5, both PT and Y59F displayed similar CD spectra characterized by β-sheet features, consistent with the predicted crystal structure (Fig. 3A). Following alkaline treatment, both proteins exhibited slight reductions in β-sheet content relative to their untreated forms, where the curve exhibited negative ellipticity around 200–205 nm (Fig. 3B). Therefore, the close similarity between PT and Y59F spectra after alkaline exposure suggests that the enhanced stability of Y59F cannot be attributed to preservation of secondary structure.

### Differential scanning calorimetry comparison of parent-type and Y59F FcγRIIIa

**Fig. 4:**
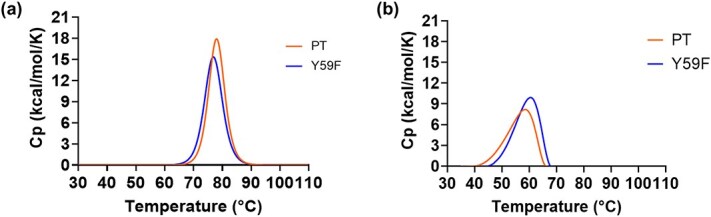
DSC analysis of PT and Y59F at (A) pH 5.5 (left) and (B) pH 12.5 (right).

To assess whether the slight disruption in β-sheet content affected overall protein tertiary structure, differential scanning calorimetry (DSC) was performed on both PT and Y59F proteins before and after alkaline treatment. In the untreated samples, both PT and Y59F exhibited comparable thermodynamic stability, with melting temperatures (*T_m_*) of 78.0 ± 0.1°C and 76.9 ± 0.2°C, and corresponding enthalpy changes of unfolding (Δ*H*_unfolding_) of approximately 127.0 ± 0.0 and 123.0 ± 1.0 kcal/mol, respectively (Fig. 4A, Table I). Following alkaline treatment, PT demonstrated a marked decrease in thermal stability, with a *T_m_* of 57.3 ± 0.2°C and Δ*H*_unfolding_ of 84 ± 0.0 kcal/mol. In contrast, the Y59F mutant retained greater stability, with a *T_m_* of 60.2 ± 0.1°C and slightly higher Δ*H*_unfolding_ of 87.6 ± 0.5 kcal/mol (Fig. 4B, Table I).

**Table I:** *T*_m_ and Δ*H*_unfolding_ of PT and Y59F at pH 5.5 and 12.5 using DSC

	*T*_m_ (°C)	Δ*H*_unfolding_ (kcal/mol)
PT, pH 5.5	78.0 ± 0.1	127 ± 1.0
Y59F, pH 5.5	76.9 ± 0.2	123 ± 0.0
PT, pH 12.5	57.6 ± 0.2	84.0 ± 0.0
Y59F, pH 12.5	60.2 ± 0.1	87.6 ± 0.5

### Tryptophan fluorescence comparison under neutral and alkaline conditions

Tyrosine has a phenolic OH with pKa ≈ 10.5 and becomes negatively charged under strongly alkaline conditions *(**24**)*. AlphaFold3 predicts two buried, spatially close tyrosines (Y59, Y72) in PT’s hydrophobic core; at pH 12.5, their simultaneous deprotonation could create electrostatic repulsion that disrupts local packing and stability (Supplementary Fig. S2). To test this, we compared wild-type PT with a conservative Y59F mutant, which removes the ionizable hydroxyl while retaining hydrophobic character.

**Fig. 5:**
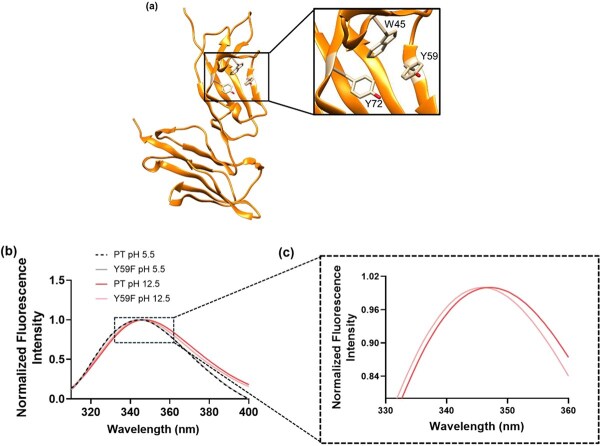
(A) AlphaFold3-predicted structure of PT highlighting buried residues W45, Y59 and Y72. (B) Tryptophan fluorescence change at pH 5.5 and pH 12.5. (C) Zoomed-in tryptophan fluorescence curve at pH 12.5 focusing on the peak top.

Conformational changes were tracked by intrinsic tryptophan fluorescence: PT has a single Trp near Y59/Y72, so its emission reports local core exposure (Fig. 5A). At pH 5.5, PT and Y59F showed virtually identical spectra, indicating an unchanged native fold. At pH 12.5, both proteins exhibited red-shifted emission consistent with increased Trp solvent exposure and partial loosening of the hydrophobic core *(**25**)*. However, PT showed a slightly larger red shift than Y59F under identical alkaline conditions (Fig. 5B and C). Although modest, this difference indicates that eliminating Tyr deprotonation via Y59F affords measurable protection against alkali-induced destabilization, supporting the proposed repulsion-driven mechanism.

### Constant pH molecular dynamics simulation at alkaline and neutral pH

Constant pH molecular dynamics (CpHMD) simulations were performed to investigate the potential electrostatic repulsion between tyrosine residues under alkaline conditions. The starting structures of PT and Y59F were predicted using AlphaFold3, with both models achieving predicted template modelling (pTM) scores of 0.86, indicating sufficient structural accuracy for molecular dynamics simulations. Furthermore, comparison of the side chain conformation between the predicted structure and crystal structure of wild-type FcγRIIIa (PDB: 5YC5) confirms that AlphaFold3 showed near identical orientation (Supplementary Fig. S2A–D). Starting with these initial structures, three independent simulations running for 300 ns each were conducted at different initial velocities. To confirm equilibration of the trajectories, we first calculated the change in root mean square deviation (RMSD) values of Cα atoms for each structure and found that each remained stable during the 300-ns simulations (Supplementary Fig. S3A and B). RMSD and root mean square fluctuation analyses of the backbone revealed no significant global structural changes in either the PT or Y59F variants (Supplementary Fig. S3C and D). Tyr protonation-state analysis (λ trajectories; λ = 0 (protonated), λ = 1 (deprotonated)) shows that Tyr59 in the PT samples fluctuates between 0 and 1, suggesting that protonation state is dynamic. Tyr72 in both PT and Y59F is predominantly deprotonated (λ ≈ 1), indicating that Tyr72 is largely ionized under the conditions modelled (Supplementary Fig. S4A–C). These results support the feasibility of sampling deprotonated tyrosine states in CpHMD and provide a framework to test whether a local electrostatic effect can explain the observed trends.

**Fig. 6:**
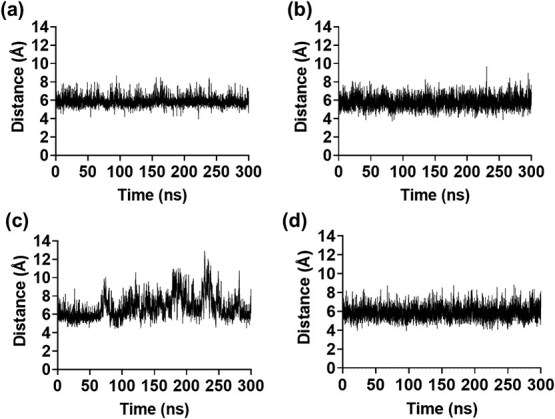
Distance changes between the ζ carbons of residues 59 and 72 over the simulation time in run 1 for (A) PT pH 7, (B) Y59F pH 7, (C) PT pH 12 and (D) Y59F pH 12.

**Fig. 7:**
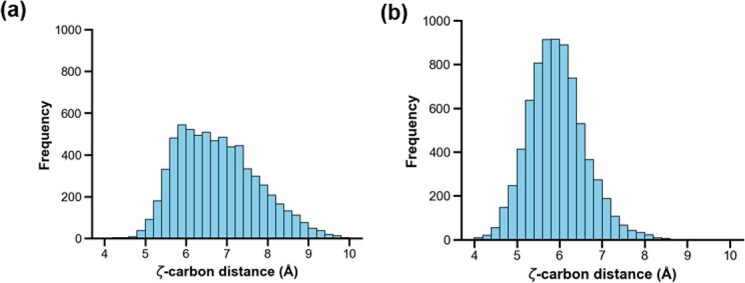
Distribution graphs showing the frequency of the distance between ζ-carbon atoms of residues 59–72 over the simulation at pH 12: (A) PT and (B) Y59F.

To observe repulsion during the simulation, the Cζ–Cζ distance between residues 59 and 72 was analysed (Supplementary Fig. S5). The results showed that both the PT and Y59F mutants maintained stable distances averaging 5.86 ± 0.47 and 5.78 ± 0.63 Å at pH 7 throughout the simulation (Fig. 6A and B). In contrast, at pH 12, PT exhibited pronounced fluctuations in the distance between Y59 and Y72 averaging 6.69 ± 0.95 Å, whereas Y59F had a similar distance of 5.83 ± 0.70 Å. Distance distribution analysis revealed that PT had a clear population shift of greater distances than that of Y59F (Figs 6C and 7A). In contrast, the Y59F mutant maintained a stable distance between F59 and Y72 under alkaline conditions (Figs 6D and 7B). Both the time-course distance plots and distance distribution histograms support our hypothesis of electrostatic repulsion between deprotonated tyrosines under alkaline conditions.

**Fig. 8:**
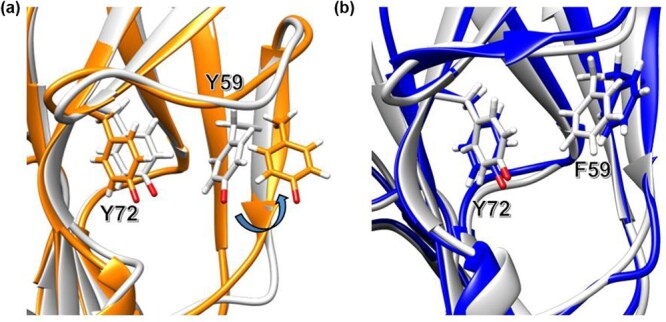
Constant pH simulation at pH 12 showing residues 59 and 72 distance changes over simulation time. (A) Structural comparison between the average structure (grey) and structure at maximum distance (orange). (B) Structural comparison of the Y59F mutant between the average structure (grey) and structure at maximum distance (blue).

These results were replicated across three independent simulations (*n* = 3), all of which revealed consistent trends (Supplementary Table S4 and Supplementary Figs S6–S8). To further investigate these observations, we analysed simulation trajectories by comparing both the average and maximum distances between the Cζ and Cζ of residues 59 and 72 with particular attention to side chain positioning. In the PT structure, the backbone near Y59 exhibited a slight outward deviation, and the Y59 side chain extended further into the solvent-exposed region, likely due to electrostatic repulsion between the deprotonated tyrosine residues under alkaline conditions (Fig. 8A). In contrast, the Y59F mutant adopted a more compact conformation, with the F59 side chain oriented slightly inward, while maintaining comparable backbone geometry (Fig. 8B).

These findings support the hypothesis that eliminating the hydroxyl group at position 59 reduces unfavourable repulsive interactions, thereby enhancing structural stability of the protein under alkaline stress.

## Discussion

The Y59F mutation in FcγRIIIa provides a compelling example of how a subtle, rational amino acid substitution can significantly improve protein resilience under harsh alkaline stress without compromising native structure or function. Our findings collectively highlight a structure–function relationship governed by local electrostatics within a sensitive region of the protein, offering mechanistic insights into pH-induced destabilization and a strategy for protein stabilization.

Under neutral conditions, Y59F displays identical secondary structure and thermal stability to PT but shows a slight reduction in functional binding by SPR and ELISA; therefore, the substitution is functionally tolerated rather than a strictly structurally conservative replacement for tyrosine at position 59. However, under alkaline conditions, the Y59F mutant exhibited significantly enhanced retention of binding activity compared to that of PT, suggesting that the mutation conserves the structure and actively protects against alkaline-induced damage.

Although DLS measurements at pH 12.5 revealed a slightly broader peak and minor increase in particles >100 nm, these findings indicate only limited aggregation and heterogeneity under alkaline conditions. Similarly, analytical SEC and SEC-MALS revealed a single, well-defined monomer peak at both pH values, and SDS-PAGE with CBB staining and western blotting confirmed protein integrity, showing no evidence of degradation or fragmentation. UV spectroscopy showed a single absorbance peak, consistent with no detectable formation of quinone or catechol. CD analysis revealed that both the PT and Y59F variants experienced partial loss of β-sheet structure upon alkaline exposure; however, the similar secondary structure of PT and Y59F under alkaline conditions suggests that the mechanism underlying Y59F’s enhanced alkaline stability is not due to stabilized secondary structure. Instead, this suggests that Y59F likely improves alkaline stability by altering the local microenvironment, such as by changing tertiary contacts or conformational flexibility.

Overall, we evaluated the major alkaline degradation pathways to identify factors that could explain the enhanced binding activity of the Y59F mutant. The only clear sign of alkaline degradation was partial unfolding with minimal aggregation, and the extent of unfolding and aggregation was indistinguishable between PT and Y59F. Chemical degradations such as deamidation, backbone cleavage and β-elimination could occur at very low levels that escape detection by bulk methods, so their complete absence cannot be formally excluded. Nevertheless, the combined biophysical and biochemical data strongly indicate that loss of chemical modifications is not the primary explanation for the improved binding of Y59F as measured by ELISA and SPR.

Bulk measures of secondary structure, aggregation and chemical integrity do not explain the functional difference, pointing instead to a localized, site-specific tertiary effect. Although PT and Y59F show similar global stability after alkaline treatment, clear differences in binding indicate that the Y59F substitution reduces alkaline-induced loss of binding competence by preventing a destabilizing local interaction under alkaline conditions.

**Fig. 9:**
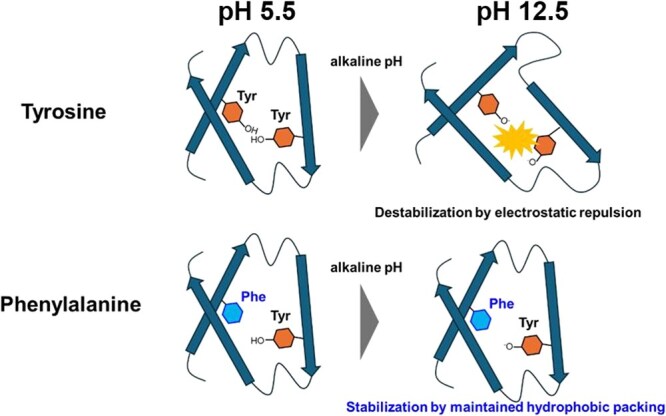
Protein destabilization by Tyr–Tyr repulsion and stabilization by Phe substitution under alkaline conditions.

We hypothesize that under alkaline conditions, the deprotonation of tyrosine side chains introduces negative charges that destabilize proteins via electrostatic repulsion, when the tyrosines are in close proximity within buried regions, a pH-dependent ionization that is a contributing factor to the local destabilization observed for proximal residues Y59 and Y72 in FcγRIIIa (Fig. 9). This is especially relevant in FcγRIIIa, where Y59 and Y72 are positioned near each other within a partially buried interface. The proximity of these residues under normal conditions is unproblematic; however, at high pH, their simultaneous deprotonation likely leads to repulsion and conformational strain. Substitution of Y59 with phenylalanine, which eliminates the hydroxyl group responsible for ionization, appeared to suppress this destabilizing interaction (Fig. 9). This interpretation is supported by multiple, convergent lines of evidence. DSC complemented these findings by demonstrating that alkaline treatment reduced the thermal stability and Δ*H*_unfolding_ of both proteins, but that PT was more severely affected.

Fluorescence spectroscopy was used to assess the tertiary structure by monitoring changes in the microenvironment of tryptophan residues. The red shift observed for both PT and Y59F mutant under alkaline conditions was indicative of increased solvent exposure, a hallmark of partial unfolding. Notably, the PT exhibited a slightly more pronounced shift, which is consistent with a greater extent of core loosening. Suppression of this shift by the Y59F mutation further supported its role in maintaining native-like packing and hydrophobic shielding under high pH stress.

Together, the red shift in tryptophan fluorescence and the decreases in *T_m_* and Δ*H*_unfolding_ point to the same structural consequence: increased solvent exposure of W45. This exposure reflects a loss of hydrophobic packing in the protein core, which reduces the number and strength of favourable noncovalent interactions and therefore lowers the enthalpic cost of unfolding. The slightly larger red shift and greater reduction in Δ*H*_unfolding_ observed for PT are consistent with more extensive core loosening and a larger destabilizing enthalpic change, whereas the attenuated red shift and smaller enthalpic loss for Y59F indicate that the mutation protects the hydrophobic core and thus maintains a higher *T*_m_ and ΔH_unfolding_ under alkaline stress. Several previous studies have reported this correlation between Trp red shifts (increased aqueous exposure) and reduced thermal stability/Δ*H*_unfolding_  *(**25**)*.

Computational analyses using constant pH molecular dynamics simulations provided mechanistic insights into this phenomenon. The predicted structure demonstrated high accuracy based on pTM scores, and structural comparison with the wild-type FcγRIIIa crystal structure showed that the overall orientation was nearly identical. This suggests that the predicted model reliably captured even buried side-chain conformations. However, our comparison relied on the co-crystal structure of FcγRIIIa bound to Fc-IgG, and the receptor conformation in this context may differ from the unbound state, representing a potential limitation of the study. While both variants remained stable at neutral pH, simulations at pH 12 revealed increased distance fluctuations between residues 59 and 72 in PT, aligning with the expectation of electrostatic repulsion between the deprotonated tyrosines. In contrast, the Y59F mutant maintained a stable inter-residue distance, indicating reduced dynamic strain. Analysis of the average and maximum distance trajectories further confirmed these observations: PT adopted conformations where the Y59 side chain extended outward, likely to relieve repulsion, whereas the Y59F side chain remained more internally oriented. Notably, these conformational shifts were subtle and localized and were not detectable by global RMSD metrics, underscoring the sensitivity of specific local interactions in determining stability outcomes.

Solvent-accessible surface area (SASA) analysis using GROMACS showed no significant difference in tryptophan solvent exposure between the pH conditions, which may reflect inherent challenges in accurately simulating subtle conformational changes under extremely alkaline conditions or limitations in the sensitivity of SASA calculations for detecting localized structural perturbations.

These results reveal an uninvestigated contributor to alkaline sensitivity in FcγRIIIa: deprotonation of buried tyrosine side chains. Previous efforts to improve alkaline stability have focused mainly on negatively charged residues such as Asp and Glu, but our data identify alkaline destabilization driven by deprotonated tyrosine as a distinct, residue-specific mechanism. A plausible, testable cause is Tyr–Tyr electrostatic repulsion following ionization of one or both buried tyrosines; this mechanism likely acts alongside other minor pathways rather than excluding them. From a protein design perspective, replacing buried tyrosine residues with phenylalanine offers a practical strategy for formulations and processes that expose proteins to alkaline conditions. Although tyrosine contributes to hydrogen bonding and structural rigidity, its susceptibility to deprotonation poses a liability in alkaline environments, whereas phenylalanine provides a near-isosteric, non-ionizable substitute that preserves hydrophobic packing and improves pH tolerance. Further work is needed to determine how generalizable this mechanism and strategy are across other proteins, but these findings point to a promising direction for developing transferable principles to engineer protein resilience. Such advances would be valuable for industrial bioprocessing and therapeutic applications that require proteins to retain stability and activity under alkaline conditions.

## Conclusion

This study demonstrated that the Y59F mutation enhances alkaline stability in FcγRIIIa by mitigating electrostatic repulsion and consequently local structural destabilization of the protein under harsh conditions. These findings highlight the utility of rational design strategies for engineering robust proteins capable of functioning in diverse, non-physiological environments.

## Materials and Methods

### Expression and purification of FcγRIIIa

The gene encoding FcγRIIIa was expressed in a pET28b vector with a 6× His tag at the C-terminus. A second construct containing an AviTag followed by a C-terminal 6× His was additionally prepared. Both plasmids were transformed into *Escherichia coli* strain BL21(DE3), and transformants were grown in 2× YT medium supplemented with 100 μg/ml kanamycin. After the culture reached an optical density of 0.6, 0.01 mM isopropyl β-d-1-thiogalactopyranoside was added and incubated for 20 h at 100 rpm and 20°C to induce protein expression. The overnight culture was harvested, and the *E. coli* pellet was resuspended in 20 mM Tris–HCl, 500 mM NaCl and 5 mM imidazole (pH 8.0), then sonicated for 15 min using an ultrasonic cell disruptor (TOMY, Tokyo, Japan). The supernatant was obtained by ultracentrifugation at 40,000 × *g* for 30 min. The supernatant was passed through a 0.8-μm filter and applied to the Ni-NTA agarose (Qiagen, Hilden, Germany) column equilibrated with 20 mM Tris–HCl, 500 mM NaCl and 5 mM imidazole (pH 8.0). The column was washed sequentially in 20 mM Tris–HCl, 500 mM NaCl and 50 mM imidazole and eluted with 20 mM Tris–HCl, 500 mM NaCl, 100 mM and 500 mM imidazole. Eluted protein was subjected to SEC using a HiLoad 16/60 Superdex 75-pg column (Cytiva, Wilmington, CA, USA) equilibrated with 50 mM sodium acetate and 500 mM NaCl (pH 5.5). The monomer peak fractions were collected for analysis. The concentration of FcγRIIIa Pt was measured by measuring absorbance at 280 nm on a UV–vis spectrophotometer and converting it to molar concentration using the molar extinction coefficient ɛ_280_ = 38,960 M^−1^ cm^−1^ calculated with the ProtParam tool (ExPASy).

### Cloning, expression and purification of Y59F FcγRIIIa mutant

Site-directed mutagenesis was performed using a three-step PCR protocol with KOD One polymerase (Toyobo, Osaka, Japan), using an FcγRIIIa plasmid as template. Following PCR amplification, the reaction mixture was treated with DpnI to digest the methylated plasmid template, and the product was purified using a column purification kit. Overall, 30 ng of purified DNA was transformed into *E. coli* JM109 cells via heat shock and plated on LB agar plates containing ampicillin. A single colony was selected and cultured in 3 ml of LB medium. Plasmid DNA was extracted using a FastGene Plasmid Mini Kit (Nippon Genetics, Tokyo, Japan). Successful cloning of the Y59F mutant was confirmed using Sanger DNA sequencing. The Y59F mutant was expressed and purified as described in Section “Expression and Purification of FcγRIIIa.” Furthermore, the concentration of FcγRIIIa Y59F mutant was measured by measuring absorbance at 280 nm on a UV–vis spectrophotometer and converting to molar concentration using the molar extinction coefficient ɛ_280_ = 37,470 M^−1^ cm^−1^ calculated with the ProtParam tool (ExPASy).

### Expression and purification of trastuzumab monoclonal antibodies

The DNA sequences encoding the heavy and light chains of trastuzumab were subcloned into the pcDNA3.4 expression vector. The vectors were co-transfected into Expi293 cells using the ExpiFectamine 293 Transfection Kit (Thermo Fisher Scientific, Waltham, MA, USA), following the manufacturer’s protocol. Transfected cells were cultured for 4 days at 37°C under 8% CO₂. After incubation, cultures were centrifuged at 2000 rpm for 10 min and the resulting supernatant was collected and passed through a 0.8-μm membrane filter. The clarified supernatant was applied to a rProtein A Sepharose Fast Flow column (Cytiva) pre-equilibrated with PBS (pH 7.4). After washing with PBS, the bound antibody was eluted with Pierce IgG Elution Buffer (Thermo Fisher Scientific) and immediately neutralized by adding 1 mM Tris–HCl (pH 8.0). The eluted fraction was further purified by SEC using a HiLoad 16/60 Superdex 200-pg column (Cytiva) equilibrated with PBS (pH 7.4). The peak fraction was collected and purity was confirmed by SDS-PAGE, which revealed a purified band of the light chain and heavy chain. Trastuzumab IgG concentration was determined from measuring absorbance at 280 nm using ProtParam calculated molar extinction coefficient ɛ_280_ = 207,200 M^−1^ cm^−1^ calculated with the ProtParam tool (ExPASy).

### ELISA of alkaline-treated and non-alkaline-treated FcγRIIIa

For alkaline-treated samples, FcγRIIIa was adjusted to a final concentration of 20 μg/ml, and NaOH was added to a final concentration of 50 mM. The mixture was incubated at 30°C for 90 min, then neutralized by adding four volumes of sodium acetate buffer (50 mM Sodium Acetate pH 5.5, 500 mM NaCl). For non-alkaline-treated controls, FcγRIIIa at 20 μg/ml was incubated at 30°C for 90 min, followed by the addition of four volumes of sodium acetate buffer without prior alkaline treatment. High-binding 96-well plates were coated with 100 μl/well of 10 μg/ml trastuzumab or 10 μg/ml BSA (as a negative control) and incubated for 16 h at 4°C for immobilization. The plates were then washed three times with PBST (PBS containing 0.05% Tween-20) and blocked with 300 μl/well of 2% (w/v) skim milk in PBST for 2 h at room temperature. After blocking, the plates were washed three times with PBST. Alkaline-treated or untreated FcγRIIIa samples (100 μl/well) were added and incubated for 1 h at room temperature. The plates were washed three times with PBST and incubated with horseradish peroxidase-conjugated anti-His antibody (MBL Life Science, Tokyo, Japan) diluted 1:15,000 in PBST for 1 h at room temperature. Following three additional washes with PBST, 50 μl/well of TMB substrate solution (Cosmo Bio, Tokyo, Japan) was added, and the reaction was allowed to proceed for 10 min in the dark. The reaction was arrested by adding 50 μl/well of stop solution, and optical absorbance was measured at 450 nm using a Pherastar microreader (BMG Labtech, Ortenberg, Germany).

### Surface plasmon resonance

For this experiment, FcγRIIIa was treated under alkaline conditions and then returned to neutral pH by measurement buffer (PBS-T, 0.05% Tween-20). Biotinylation of FcγRIIIa mutants containing a C-terminal Avi-tag was performed using a BirA Biotinylation kit (Cosmo Bio). Following biotinylation, proteins were dialysed overnight against SPR measurement buffer to remove unreacted BirA and excess biotin. After dialysis, samples were either alkaline treated or left untreated, and then diluted to a final concentration of 100 nM. SPR measurements were performed on a Biacore S200 instrument (Cytiva) using a streptavidin (SA) sensor chip. Biotinylated FcγRIIIa (treated or untreated) was immobilized to a target level of 50 RU. Trastuzumab was prepared in measurement buffer at concentrations of 400, 200, 100, 50 and 25 nM and injected as the analyte. Surface regeneration was performed using 1 M arginine hydrochloride, pH 4. All measurements were done three times, the kinetic parameters were averaged and the standard deviation was taken as uncertainty.

### Aggregation measurement by UNcle

DLS measurements were performed using the UNcle platform (Unchained Labs) at 25°C. In this experiment, FcγRIIIa samples that had been exposed to alkaline conditions were measured directly at the alkaline pH; untreated controls were measured in parallel. Both alkaline-treated and untreated FcγRIIIa samples were adjusted to a concentration of 1 mg/ml in sodium acetate buffer. Measurements were performed in triplicate for each condition. The mass distribution and hydrodynamic radius of each sample were analysed at 25°C, and data were processed using UNcle analysis software according to the manufacturer’s guidelines.

### Analytical SEC

Analytical SEC was performed using a Superdex 200 Increase 10/300 GL column (Cytiva) connected to an ÄKTA Pure chromatography system (Cytiva). The column was equilibrated and run with a buffer containing 50 mM sodium acetate (pH 5.5) and 500 mM NaCl at a flow rate of 0.5 ml/min at 4°C. Alkaline-treated samples were neutralized by an addition of 4× volume of SEC buffer and, in parallel with untreated samples, were prepared to a final concentration of 5 μM and injected onto the column. Elution profiles were monitored to assess the SEC distribution of each sample.

### Size exclusion chromatography–multi-angle light scattering

Alkaline-treated samples were incubated then neutralized similarly to analytical SEC and, in parallel with untreated samples, were prepared to a final concentration of 1 mg/ml. The molecular weight of complexes was determined using Superdex 200 Increase 10/300 GL column (Cytiva) connected to a DAWN8+ MALS (Wyatt Technology, Santa Barbara, CA, USA), an ultraviolet detector (Shimadzu, Tokyo, Japan), and a refractive index detector (Shodex, New York, NY, USA). Protein samples (100 μl; NaOH-treated and untreated samples) were prepared to a final concentration of 42 μM (1 mg/ml). Analysis was performed using ASTRA software (Wyatt Technology). The protein concentration was calculated from the refractive index. All detectors were calibrated using bovine serum albumin (Sigma-Aldrich, St Louis, MO, USA).

### SDS-PAGE and western blotting

Protein samples were mixed with sample buffer containing Tris, SDS, glycerol, β-mercaptoethanol and bromophenol blue, and boiled at 90°C for 3 min. The denatured proteins were separated on a 15% polyacrylamide gel at 200 V for 60 min. One gel was stained with CBB solution and subsequently destained using Milli-Q water. The other gel was used for western blotting: proteins were transferred onto an Amersham Protran Premium NC 0.45 μm nitrocellulose membrane (Cytiva) using a semi-dry transfer system at 100 mA for 40 min.

The membrane was blocked with 2% (w/v) skim milk in PBST for 1 h at room temperature, followed by incubation with an anti-His-tag monoclonal antibody conjugated to HRP (mAb-HRP-DirecT; MBL Life Science) at a 1:15,000 dilution for 1 h at room temperature. Signal detection was performed using enhanced chemiluminescence reagents (Cytiva), and images were acquired using an Amersham Imager 600 (GE Healthcare, Chicago IL, USA).

### Protein concentration assay before and after alkaline treatment

Untreated (pH 5.5) and alkaline-treated (pH 12.5) FcγRIIIa samples (1.0 mg/ml) were incubated at 30°C for 30, 60 and 90 minutes (three independent replicates per variant). After each incubation, samples were filtered through a 0.2-μm membrane and the protein concentration of the soluble fraction was measured by UV–vis spectrophotometry using a Thermo Scientific NanoDrop One (Thermo Fisher Scientific). Absorbance values at 280 nm were converted to milligrams per millilitre using molar extinction coefficients calculated with ProtParam.

### UV spectroscopy

UV–vis spectra were recorded on a JASCO V-660 spectrophotometer (JASCO, Hachioji, Japan) using a 1.0-mm quartz cuvette. Alkaline-treated FcγRIIIa samples were measured directly at the alkaline pH (no neutralization); untreated controls were measured in parallel. Samples were prepared at 0.5 mg/ml in 50 mM sodium acetate and 500 mM NaCl, and equilibrated to 25°C prior to measurement. A buffer blank was recorded for each condition and subtracted from the protein spectra. Spectra were acquired from 200 to 500 nm with a bandwidth of 1 nm and a scan speed of 200 nm/min; each spectrum is the average of three independent replicate measurements. Data were processed using the instrument software (baseline correction and smoothing).

### CD spectroscopy

 CD analysis was performed using a JASCO J-820 spectropolarimeter (JASCO, Hachioji, Japan). For measurements in the far-ultraviolet region, alkaline-treated samples were analyzed directly at pH 12.5 (50 mM sodium acetate, 500 mM NaCl, 50 mM NaOH), whereas non-treated samples were measured at pH 5.5 (50 mM sodium acetate, 500 mM NaCl). All samples were prepared at a concentration of 30 μM and placed in 0.1 mm quartz cells. Each sample was measured in triplicate; the JASCO software automatically averaged the three scans, and the averaged spectrum was used to calculate the molar ellipticity (*26*):


\begin{align*} \textrm{Mol}.\ \textrm{Ellip}. = 100\times\theta/(C\times\ d) \end{align*}

where *θ* is the observed ellipticity (°), *C* is the concentration of the protein (M) and *d* is the path length (cm).

### Differential scanning calorimetry

DSC measurements were performed using a MicroCal PEAQ-DSC Automated System (Malvern, Worcester, UK). The protein samples (0.5 mg/ml in 50 mM sodium acetate, 500 mM NaCl, pH adjusted using NaOH) were either prepared by alkaline treatment (addition of NaOH) or left untreated. Alkaline-treated samples were measured directly at the alkaline pH without neutralization, and untreated samples were measured under native buffer conditions. Samples were heated from 10°C to 110°C at a scanning rate of 1.0°C/min for NaOH treatment. Each condition was measured in three independent replicates. Data were analysed using MicroCal PEAQ-DSC software (Malvern).

### Tryptophan fluorescence

Intrinsic tryptophan fluorescence was measured to evaluate conformational changes upon alkaline treatment. Protein samples (30 μM; ~0.5 mg/ml) were prepared in 50 mM sodium acetate buffer containing 500 mM NaCl, with pH adjusted according to treatment conditions, and measured immediately after treatment. Fluorescence spectra were recorded at room temperature using Fluorescence Spectrophotometer F-2500 (Hitachi, Tokyo, Japan) equipped with a deuterium lamp, using a 1-cm quartz cuvette. Emission spectra (300–400 nm) were collected at an excitation wavelength of 295 nm with 5-nm excitation and emission slit widths. Each sample was scanned three times and averaged. Buffer spectra were recorded and subtracted as background. Spectra were normalized to the maximum intensity to compare emission maxima (λmax). Blue and red shifts were interpreted as decreased and increased solvent exposure of tryptophan residues, respectively.

### Constant pH simulations of FcγRIIIa at different pHs

CpHMD simulations of FcγRIIIa were performed at different pH values. The initial structure of the FcγRIIIa mutant was predicted using AlphaFold 3 and used as the starting model for all simulations (*27*). CpHMD simulations were conducted using GROMACS 2022.1 with the CHARMM36m force field (*28*–*31*). System setup for constant pH conditions was performed using phbuilder (*32*). In addition to standard titratable residues, tyrosine residues were allowed to sample protonated and deprotonated states using modified CHARMM36m parameters (*33*). The system was solvated with TIP3P water, neutralized, energy minimized and equilibrated under NVT and NPT conditions. Production simulations were carried out in three independent runs of 300 ns. Detailed simulation protocols and parameter modifications are described in the Supporting Information.

## Supplementary data

Supplementary Data are available at *JB* Online.

## Supplementary Material

Web_Material_mvag023
