# Supplementary material for: Mitigating alkaline instability induced by tyrosine–tyrosine repulsion in an FcγRIIIa-binding protein through phenylalanine substitution
Source: J Biochem. 2026 Mar 13;179(5):317–28. doi: 10.1093/jb/mvag023 (PMC13112020; doi:10.1093/jb/mvag023)
Supplement: Web_Material_mvag023 [file web_material_mvag023.docx]

Supplementary Information

# **Title**

**Mitigating Alkaline Instability Induced by Tyrosine–Tyrosine Repulsion in an FcγRIIIa-Binding Protein Through Phenylalanine Substitution^[[1]](#footnote-1)^**

**Authors**

Rio Okuda ^1^, Yuki Tokunaga ^1^, Satoru Nagatoishi ^1,2^*, Ryo Matsunaga ^1^, Yosuke Terao ^3^, Teruhiko Ide ^3^, Kouhei TSUMOTO ^1,2,4^*

**Affiliations**

^1^ Department of Bioengineering, School of Engineering, The University of Tokyo, 7-3-1 Hongo, Bunkyo-ku, Tokyo 113-8656, Japan.

^2^ Medical Device Development and Regulation Research Center, School of Engineering, The University of Tokyo, 7-3-1 Hongo, Bunkyo-ku, Tokyo 113-8656, Japan.

^3^ Life Science Research Laboratory, Tosoh Corporation, 2743-1 Hayakawa, Ayase, Kanagawa, Japan.

^4^ The Institute of Medical Science, The University of Tokyo, 4-6-1 Shirokanedai, Minato-ku, Tokyo, 108-8639, Japan.

*** Correspondence and requests for materials should be addressed to**

S.N. ([s-nagatoishi@g.ecc.u-tokyo.ac.jp](file:///C:\Users\Zamzam%20Abou%20El%20Ella\Desktop\OneDrive%20-%20CACTUS\Jobs\FFGPW_5\s-nagatoishi@g.ecc.u-tokyo.ac.jp)) and K.T. ([tsumoto@bioeng.t.u-tokyo.ac.jp](mailto:tsumoto@bioeng.t.u-tokyo.ac.jp))

**Supplementary Method**

The initial structure of the FcγRIIIa mutant was predicted using AlphaFold 3, a state-of-the-art deep learning model for biomolecular structure prediction. The full-length FcγRIIIa amino acid sequence was submitted to the AlphaFold 3 server, and the resulting 3D model was used as the starting structure for all subsequent simulations. Structural confidence was evaluated using AlphaFold pTM scores. CpHMD simulations were conducted using GROMACS 2022.1 with the CHARMM36m force field. The system setup for constant pH conditions was performed using phbuilder, facilitating the identification and parameterization of titratable residues for CpHMD simulations in GROMACS**.** In addition to standard titratable residues (Asp, Glu, His, and Lys), Tyr residues were modified to enable the sampling of their deprotonated states using customized parameters to support the pH-dependent ionization of tyrosine. Information concerning the electronic states of protonated and deprotonated tyrosine residues was obtained from (Sobieraj M et al., 2015) where they have set the pKa of tyrosine as 10.6. Furthermore, within the CHARMM36m force field, several parameter files were modified accordingly, The merged.rtp file was updated to include a deprotonated tyrosine residue and to assign atom charges consistent with the electronic state described in (Sobieraj M et al., 2015). The merged.hdb file was adjusted to ensure correct hydrogen-building rules for the deprotonated form, and the residuetypes.dat file was updated to register the new deprotonated tyrosine as a distinct residue type. This system was solvated with the TIP3P water model, neutralized with counterions, and energy-minimized using the steepest descent algorithm for up to 5,000 steps (convergence criterion: maximum force < 1,000 kJ mol⁻¹ nm⁻¹). Equilibration was performed under NVT and NPT ensembles with position restraints on protein heavy atoms for 200 ps. The temperature was raised from 50 to 298 K over 150 ps by simulated annealing and maintained at 298 K using a velocity-rescale thermostat during NVT equilibration, while the pressure was controlled at 1 bar with an isotropic C-rescale barostat during NPT equilibration. The protonation state exchange of titratable residues, including tyrosine, was enabled throughout the simulation. Production simulations were performed in three independent runs for 300 ns and trajectory data were collected after equilibration. The structural and protonation dynamics were analyzed using standard GROMACS tools, with further inspection of tyrosine ionization behavior across the trajectory.

Supplementary Table 1. Optical absorbance was measured at 450 nm for each well and values were corrected by subtracting BSA negative control values

| **PT pH 5.5** | **Y59F pH 5.5** | **PT pH 12.5** | **Y59F pH 12.5** |
| --- | --- | --- | --- |
| 2.00 | 1.44 | 0.23 | 0.99 |
| 1.77 | 1.56 | 0.24 | 0.86 |
| 1.87 | 1.38 | 0.15 | 0.79 |
| 1.87 | 1.57 | 0.25 | 0.88 |
| 1.88 | 1.49 | 0.22 | 0.88 |

Supplementary Table 2. Kinetic Parameters obtained from SPR

|  | k_on_ (x10^5^ 1/Ms) | k_off_ (x10^-2^ 1/s) | K_D_ (nM) |
| --- | --- | --- | --- |
| PT, pH 5.5 | 3.31 ±0.09 | 3.05 ± 0.11 | 90.11 ± 1.21 |
| Y59F, pH 5.5 | 3.23 ± 0.14 | 3.25 ± 0.13 | 100.23 ± 0.68 |
| PT, pH 12.5 | n.d. | n.d. | n.d. |
| Y59F, pH 12.5 | 3.71 ± 0.01 | 3.37 ± 0.08 | 90.40 ± 1.14 |

*n.d, not determined.

Supplementary Table 3. Protein concentration before and after alkaline treatment in mg/ml

|  | 0 min | 30 min | 60 min | 90 min |
| --- | --- | --- | --- | --- |
| PT, pH 5.5 | 0.988 ± 0 | 0.992 ± 0.006 | 0.994 ± 0.014 | 0.989 ± 0.008 |
| Y59F, pH 5.5 | 0.977 ± 0 | 0.995 ± 0.003 | 0.988 ± 0.007 | 0.990 ± 0.007 |
| PT, pH 12.5 | 0.998 ± 0 | 0.994 ± 0.008 | 0.992 ± 0.006 | 0.989 ± 0.008 |
| Y59F, pH 12.5 | 0.997 ± 0 | 0.990 ± 0.007 | 0.980 ± 0.006 | 0.980 ± 0 |

Supplementary Table 4. Average distance between residue 59 and 72 throughout the CpHMD simulation (N = 3): summary of Run 2 and Run 3

|  | Run 2 (Å) | Run 3 (Å) |
| --- | --- | --- |
| PT, pH 7 | 5.87 ± 0.47 | 5.96 ± 0.53 |
| Y59F, pH 7 | 5.80 ± 0.66 | 5.96 ± 0.67 |
| PT, pH 12 | 6.72 ± 1.22 | 6.56 ± 1.00 |
| Y59F, pH 12 | 5.94 ± 0.66 | 5.85 ± 0.65 |

**Figure Legend**

Supplementary Figure 1. Mass distribution (A) and (B) intensity distribution analysis of PT (orange) and Y59 (blue) at pH 5.5 (solid line) and pH 12.5 (dotted line) using DLS. Analytical SEC comparison of NaOH-treated PT (C) and Y59F (D) at pH 5.5 and 12.5. PT (orange), Y59F (blue), pH 5.5 (solid line), and pH 12.5 (dotted line). SEC-MALS of PT pH 5.5 (E), pH 12.5 (F), Y59F pH 5.5 (G), pH 12.5 (H) and the molecular weight distribution is shown as a black solid line. (I-L) SDS-PAGE with CBB staining and western blot analysis of PT and Y59F after NaOH treatment where (I) CBB staining of PT, (J) CBB staining of Y59F, (K) western blot of PT, (L) western blot of Y59F, (M) UV spectroscopy of PT and (N) UV spectroscopy of Y59F

Supplementary Figure 2. Structural comparison of AlphaFold3 Predicted Structure and Co-crystal structure of Wild Type FcγRIIIa (PDB ID: 5YC5) (A) Full-scale comparison between PT (Orange) and Crystal Structure (Grey), (B) Full-scale comparison between Y59F (Blue) and Crystal Structure (Grey), (C) Specific comparison of residues 50 and 72 between PT and Crystal Structure, and (D) Specific comparison of residues 50 and 72 between Y59F and Crystal Structure.

Supplementary Figure 3. RMSD (Cα) and RMSF (backbone) analysis of constant pH MD simulations of PT and Y59F mutants over three 300 ns runs: (A) RMSD at pH 7, (B) RMSD at pH 12, (C) RMSF at pH 7, and (D) RMSF at pH

Supplementary Figure 4. Protonation state analysis of (A) PT Phe59, (B) PT Tyr72, (C) Y59F Tyr72

Supplementary Figure 5. Crystal structure of PT highlighting Cζ (orange) positions of residues Y59 and Y72 (blue arrow) for inter-residue distance analysis (black arrow; same carbon position was analyzed in Y59F).

Supplementary Figure 6. Analysis of trajectories measuring the distance between sidechain residues at positions 59 and 72 over time at pH 7.

Supplementary Figure 7. Analysis of trajectories measuring distance between sidechain residues at positions 59 and 72 over time at pH 12.5.

Supplementary Figure 8. Distribution graphs showing frequency of the distance between ζ-carbon atoms of residues 59–72 over the simulation at pH 12.


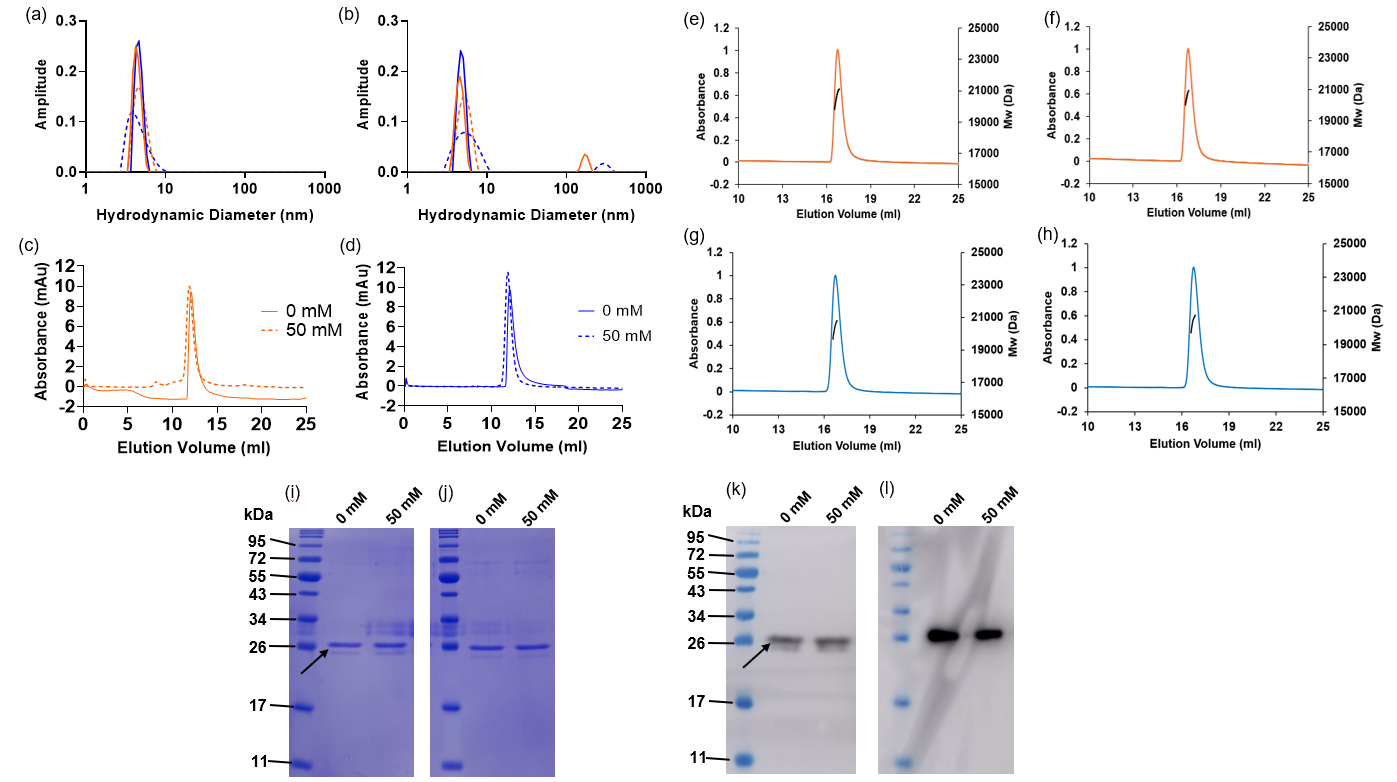


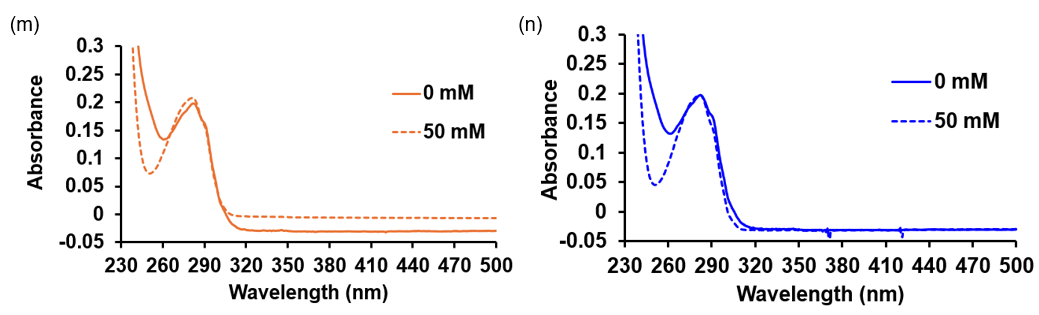


Supplementary Figure 1


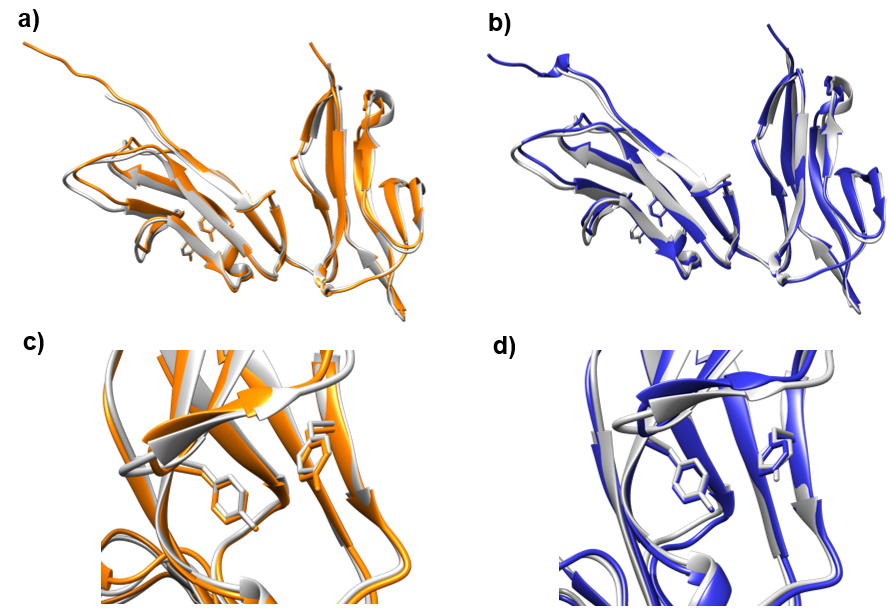


Supplementary Figure 2


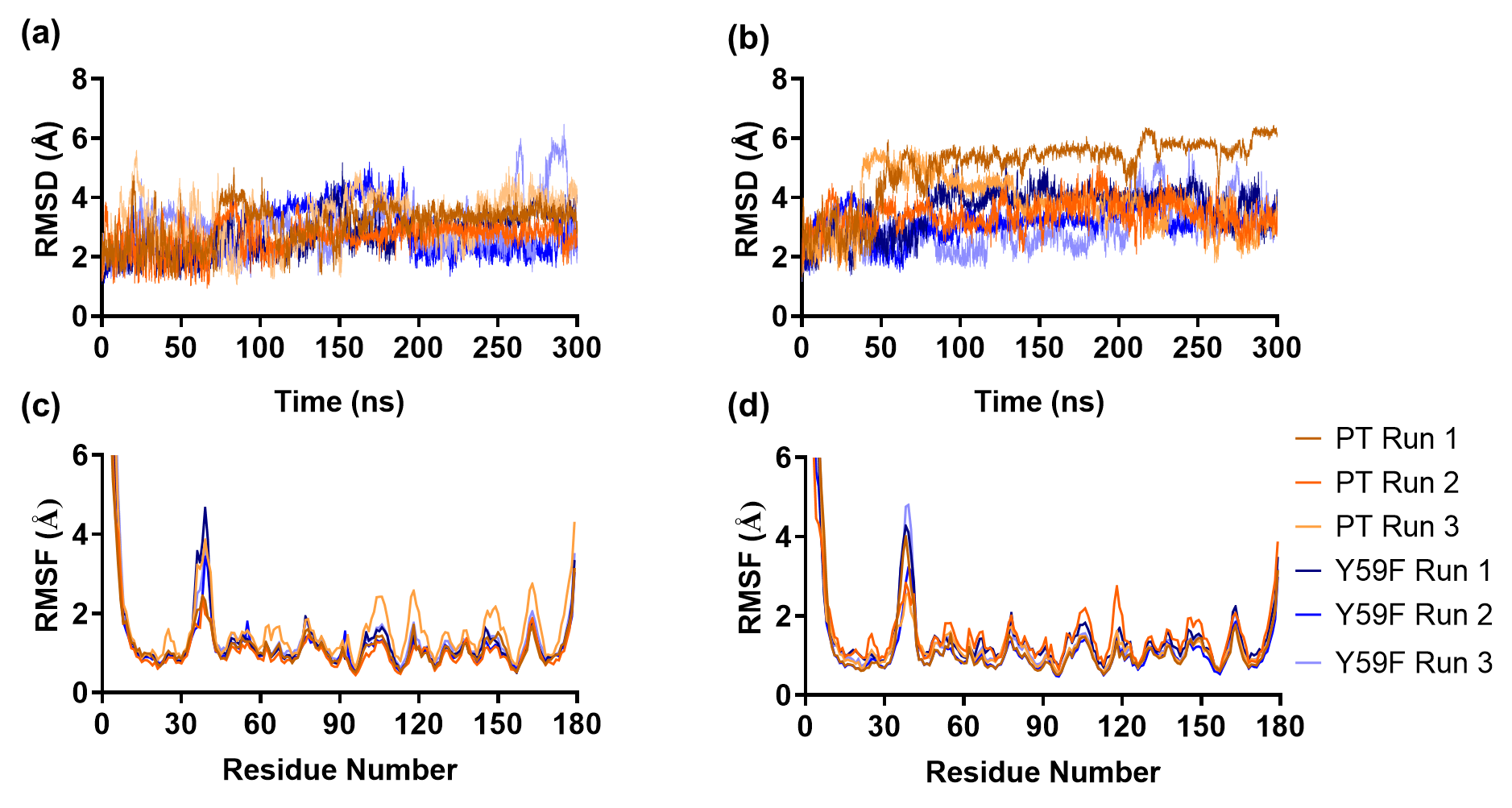


Supplementary Figure 3


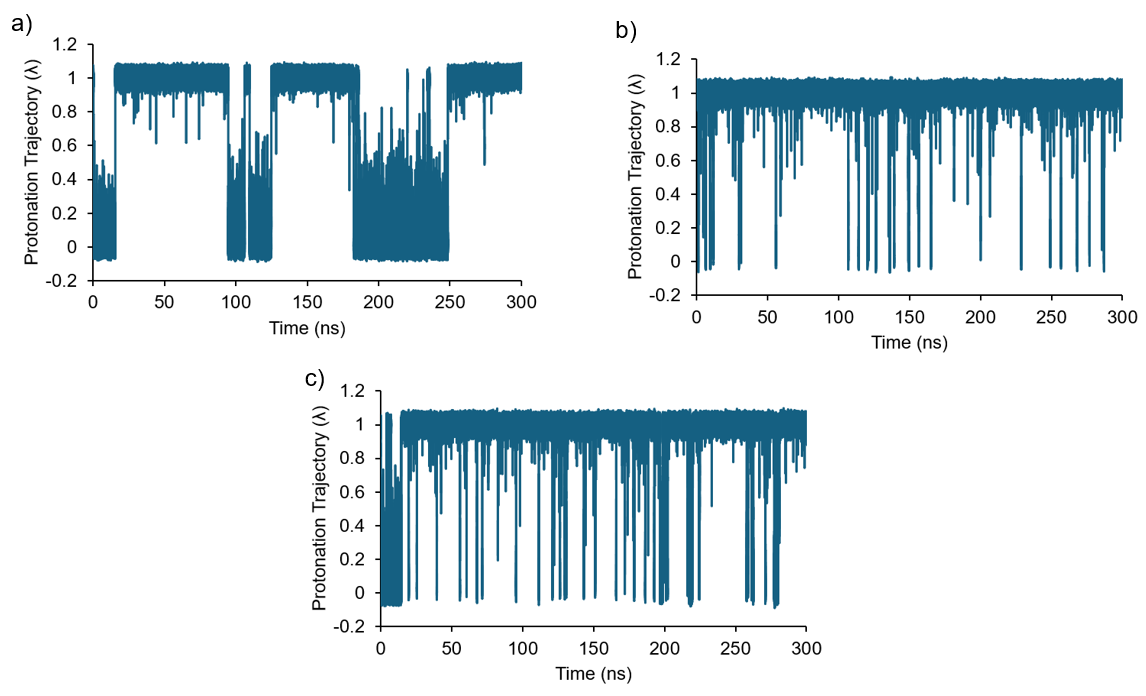


Supplementary Figure 4


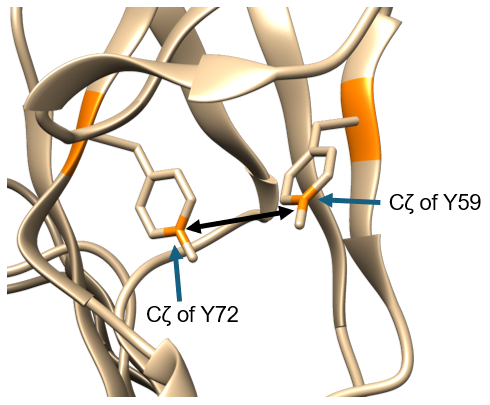


Supplementary Figure 5


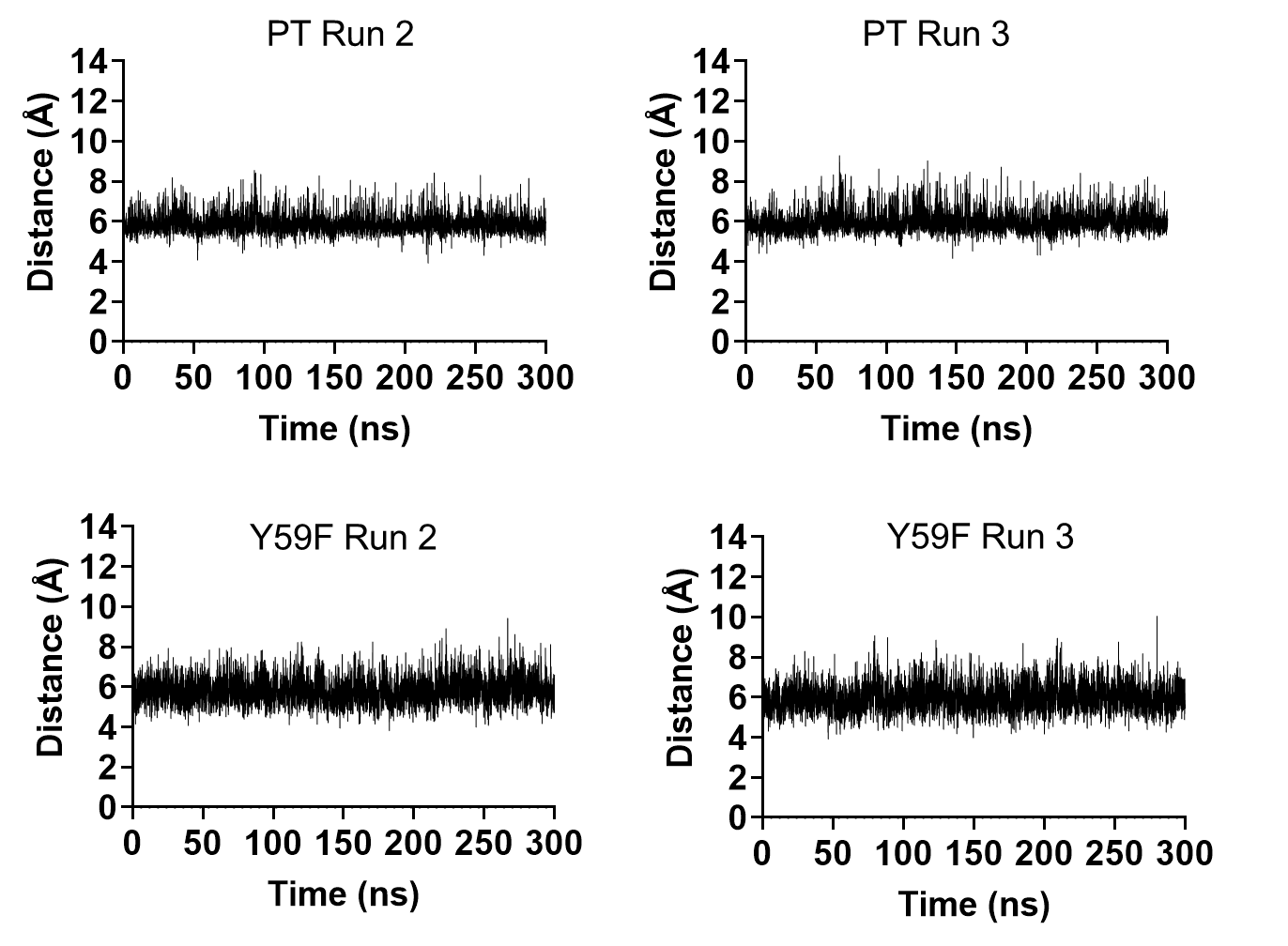


Supplementary Figure 6.


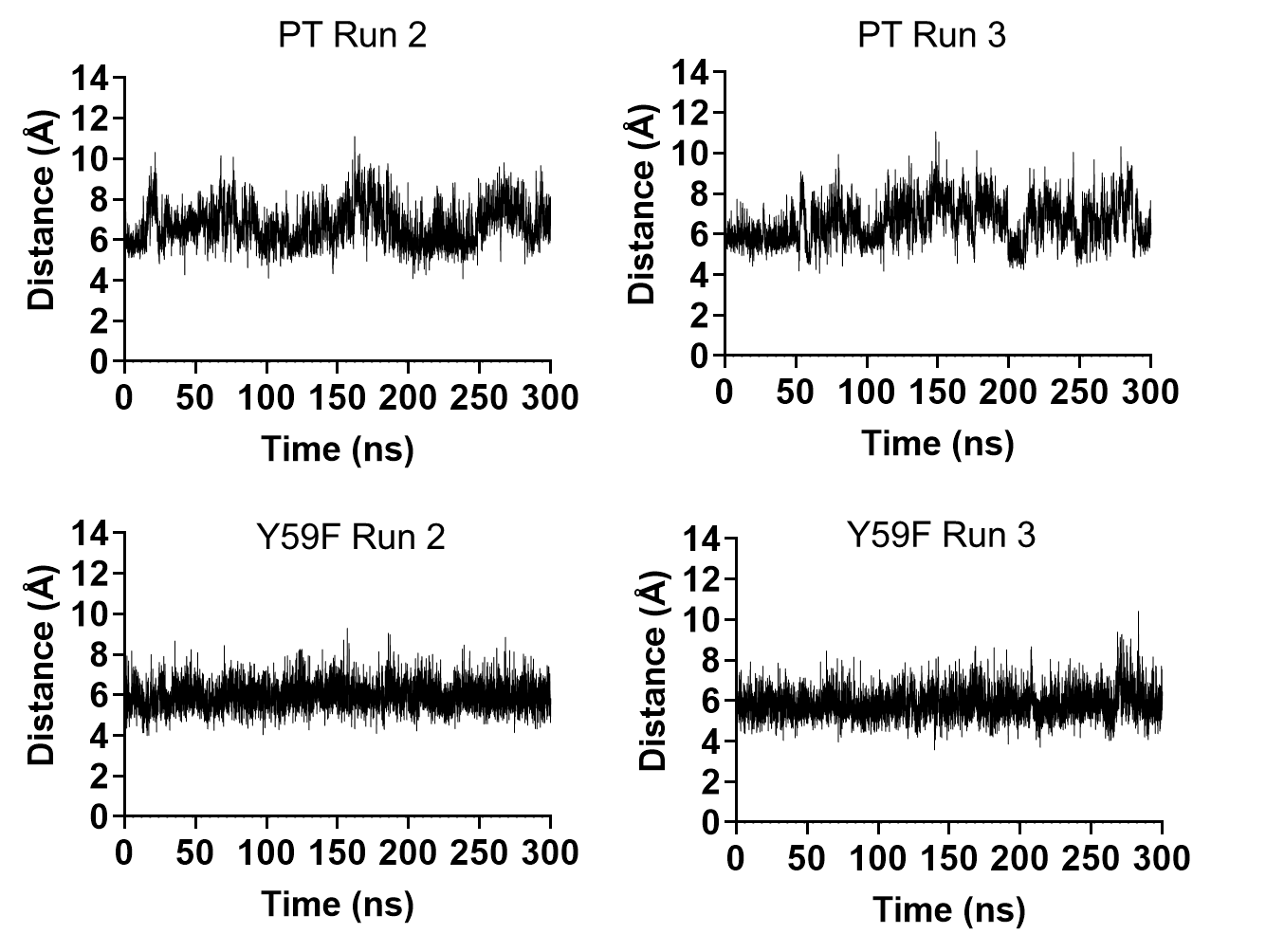


Supplementary Figure 7


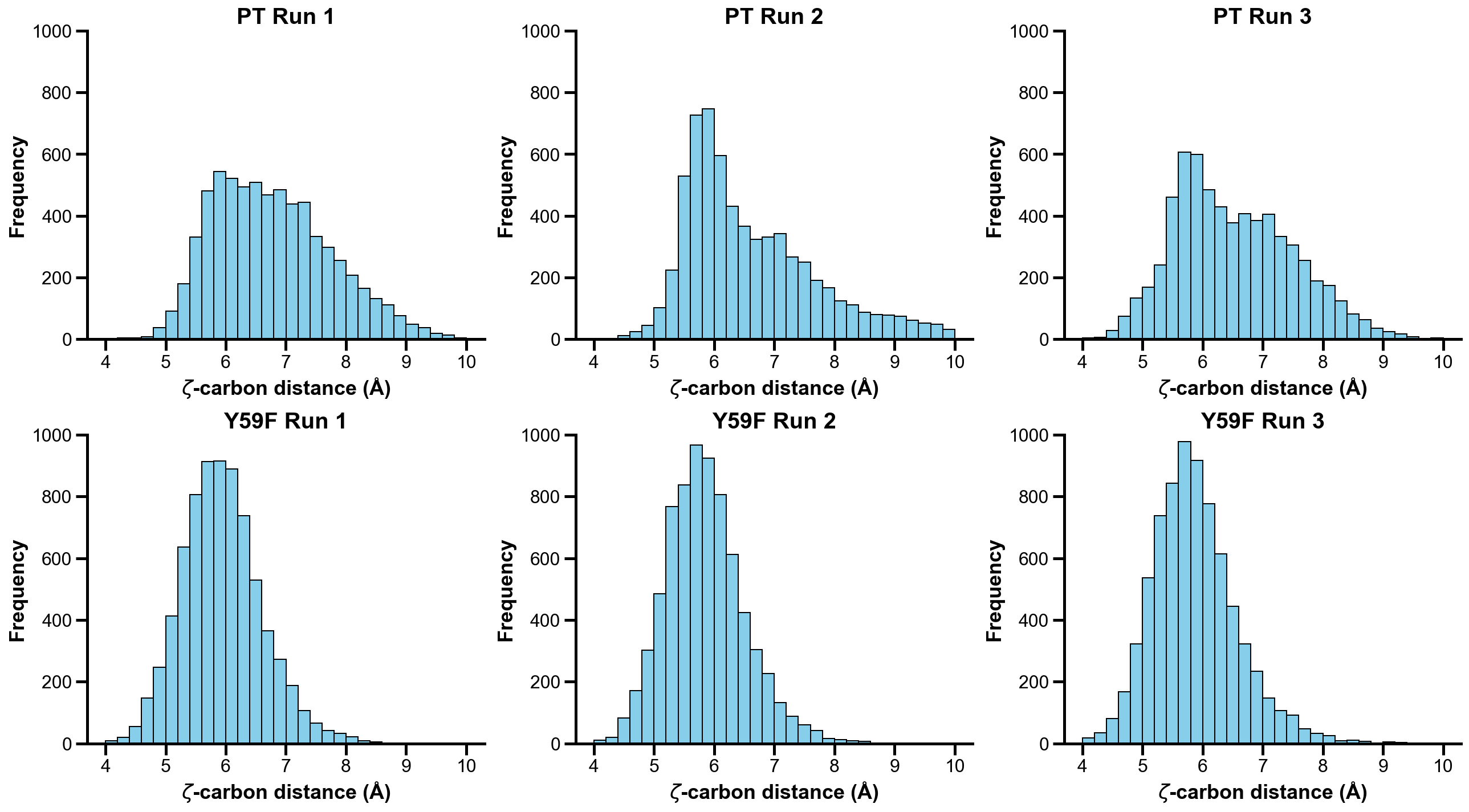


Supplementary Figure 8

# **Glosssary**

CIP, clean-in-place; FcγRIIIa, Fc gamma receptor IIIa; ELISA, enzyme-linked immunosorbent assay; Y59F, substitution of tyrosine at position 59 with phenylalanine; PT, parent-type FcγRIIIa; SEC, size-exclusion chromatography; CBB, Coomassie brilliant blue; DLS, dynamic light scattering; CD, circular dichroism; DSC, differential scanning calorimetry; Tm, melting temperature; ΔHunfolding, enthalpy changes of unfolding; CpHMD, constant pH molecular dynamics; RMSD, root mean square deviation; pTM, predicted Template Modeling; SASA, solvent-accessible surface area; SEC-MALS, Size exclusion chromatography-multi-angle light scattering.

1. [↑](#footnote-ref-1)
